# First Derivative UV Spectra of Surface Water as a Monitor of Chlorination in Drinking Water Treatment

**DOI:** 10.1100/tsw.2001.13

**Published:** 2001-04-04

**Authors:** V. Zitko

**Affiliations:** Fisheries and Oceans Canada, Marine Environmental Sciences, St. Andrews, New Brunswick E5B 2L9, Canada

**Keywords:** principal component analysis, chlorination, natural organic matter, humic acid, fulvic acid, lake water

## Abstract

Many countries require the presence of free chlorine at about 0.1 mg/l in their drinking water supplies. For various reasons, such as cast-iron pipes or long residence times in the distribution system, free chlorine may decrease below detection limits. In such cases it is important to know whether or not the water was chlorinated or if nonchlorinated water entered the system by accident. Changes in UV spectra of natural organic matter in lakewater were used to assess qualitatively the degree of chlorination in the treatment to produce drinking water. The changes were more obvious in the first derivative spectra. In lakewater, the derivative spectra have a maximum at about 280 nm. This maximum shifts to longer wavelengths by up to 10 nm, decreases, and eventually disappears with an increasing dose of chlorine. The water treatment system was monitored by this technique for over 1 year and changes in the UV spectra of water samples were compared with experimental samples treated with known amounts of chlorine. The changes of the UV spectra with the concentration of added chlorine are presented. On several occasions, water, which received very little or no chlorination, may have entered the drinking water system. The results show that first derivative spectra are potentially a tool to determine, in the absence of residual chlorine, whether or not surface water was chlorinated during the treatment to produce potable water.

## INTRODUCTION

Chlorination is a widely used disinfection process in the production of drinking water. In addition, the presence of a low concentration of free chlorine ("residual chlorine") protects drinking water in the distribution system. The residual chlorine, legally required in many countries, is usually monitored at the outflow of the treatment plant and at several points in the distribution system. The absence of free chlorine may indicate a potential public health problem. It may be that the residual chlorine was decomposed in the distribution system or that, by accident, the water was not chlorinated at all. In particular, the latter may result in a serious public health problem. It is therefore important, in the absence of residual chlorine, to be able to determine the chlorination "history" of drinking water. This paper shows that changes in the UV spectra of natural organic matter (NOM, dissolved organic carbon, fulvic acid, humic acid) present in surface water may be used for this purpose.

NOM of surface waters has relatively featureless UV spectra, with a small maximum or a shoulder at about 280 nm. In general, the absorbance increases almost monotonically with decreasing wavelength[1]. UV absorbance, usually at 254 nm, has been used to estimate the concentration of total organic carbon. The UV absorbance is caused by aromatic structures in the NOM molecules and, consequently, decreases with the destruction of the aromatic structures by chlorination[2-4]. The decrease in UV absorbance is correlated to the formation of organic halogen compounds and disinfection by-products. This correlation is improved by determining the absorbance difference between original and chlorinated water samples[5].

In this work, the drinking water system of a small town was studied. The town obtains its water from a lake. The water is chlorinated at the lake, pumped to a storage reservoir, and from there distributed to one large-volume user for primarily nondrinking applications, and to the town drinking water system. As a rule, no free chlorine was detectable in the water received by the large-volume user during the period of the study. Before entering the town drinking water system, the water is rechlorinated. After the rechlorination, the concentration of free chlorine is usually about 0.2–0.5 mg/l, but may fluctuate from 0 to over 1 mg/l. Since no free chlorine was detectable in the large-volume users' system, and, occasionally, in the town system, it was important, for reasons of public health, to determine whether or not the water was chlorinated at the lake. First derivative UV spectra were used for this purpose.

## MATERIALS AND METHODS

Water samples were taken periodically from February 1998 until August 1999 at the "large-volume user" (U) and at the "town" (T) station. The UV spectra were recorded either within a few hours of collection, or the samples were stored at 5°C for a maximum of 4 d.

Free chlorine was measured by the DPD method[6]. A Hewlett-Packard Diode Array spectrophotometer Model 8452A with a 2-nm resolution was used to scan the spectra from 200 to 350 nm. First derivatives were calculated according to Savitzky and Golay[7] by the "savgol" method of MATLAB (The MathWorks Inc., Natick, MA 01760, USA). An 11-points window and a quadratic polynomial were used in the calculations.

For a graphical presentation of a large number of spectra, sections of the first derivative spectra (240–320 nm) were, without any preprocessing, analyzed by Principal Component Analysis (PCA) software[8], and the projection on the plane of the first two principal components, which accounted for 99.93% of the original sums of squares, was examined.

To determine the effect of chlorination on the UV spectrum of lakewater, 0.5, 1, 2, and 4 ml of a commercial bleach (5.7% chlorine, diluted 100 times) were added to 1 l of lakewater, to yield initial chlorine concentrations of 0.27, 0.55, 1.11, and 2.22 mg/l, respectively. The spectra were recorded after 2 and 24 h.

## RESULTS AND DISCUSSION

Chlorination causes a gradual disappearance of a maximum in the first derivative spectrum of the lakewater (Fig. 1). Even at an initial chlorine concentration of 2.22 mg/l, the first derivative spectrum (5) is still distinguishable from the average spectrum (6) of the monitoring period. However, after 24 h, the first derivative spectrum of sample that received an initial chlorine concentration of 2.22 mg/l, becomes much more similar to the average spectrum (Fig. 2). The spectrum of the sample with initial chlorine concentration of 1.11 mg/l has changed slightly. Spectra of samples with still lower initial chlorine concentrations (1,2, Fig. 1) remained practically unchanged after 24 h (not shown).

It can be seen from the difference between the average first derivative spectrum and the spectrum of the sample with initial chlorine concentration of 2.22 mg/l (Fig. 1, spectra 6 and 5, respectively) that, **on the average**, water in the system received initially more chlorine than 2.22 mg/l. Alternatively, according to the appearance of the first derivative spectra, scanned after 2 and 24 h (Fig. 2), the water may have been retained in the system for at least 24 h before sampling, but **it was initially chlorinated**.

Spectra from approximately 1.5 years of monitoring, projected on the plane of the first two principal components are summarized in Fig. 3. The spectra show a relatively large variation along the first principal component axis (pc-1). The three clusters visible along this axis appear to be related to the season. Those in the right-hand cluster were obtained mostly in the spring of 1998, those in the central cluster, during 1999, and those in the left-hand cluster, during summer, fall, and winter of 1998. Water levels in the lake, and maintenance work on some of the water lines in the system during the monitoring period, may be responsible for these differences.

As indicated by the locations of lakewater samples, chlorinated in the laboratory (Fig. 3), the initial chlorine concentration affects the position of the spectra along the 2$^{nd}$ principal component (pc-2) axis. This is shown also in Fig. 4 by plotting the pc-2 scores against the initial concentration of chlorine. At higher chlorine concentration, the spectra change further during 24 h after the addition of chlorine, as mentioned earlier. However, it is the samples with high positive pc-2 scores that are of particular interest, since they correspond to water that received little chlorine. As can be seen from Fig. 3, the "large-volume user" samples (U) predominate at positive pc-2 values, since this water is not rechlorinated. Interestingly, also the town system (T) received, at times, water with a low initial concentration of chlorine. First derivative spectra of such samples from both, the large-volume user and the town, are shown in Fig. 5.

## CONCLUSIONS

It appears that first derivative spectra of surface waters may be used to assess qualitatively the chlorination "history" of such samples, which is important when no residual chlorine is detectable. This technique suggests, that on several

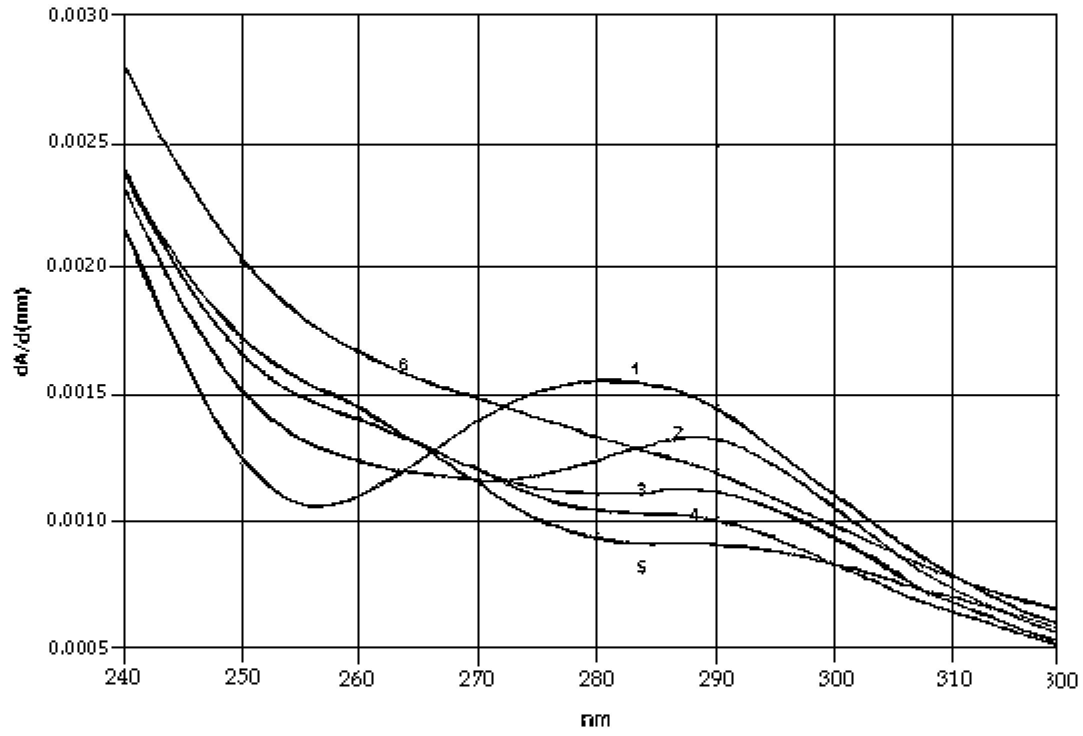

**FIGURE 1**. First derivative UV spectra of lake water and chlorinated lakewater, measured 2 h after the addition of chlorine. Initial chlorine concentrations, mg/l: 0 (1), 0.27 (2), 0.55 (3), 1.11 (4), and 2.22 (5). The first derivative of the average spectrum of all water samples is included for comparison (6).

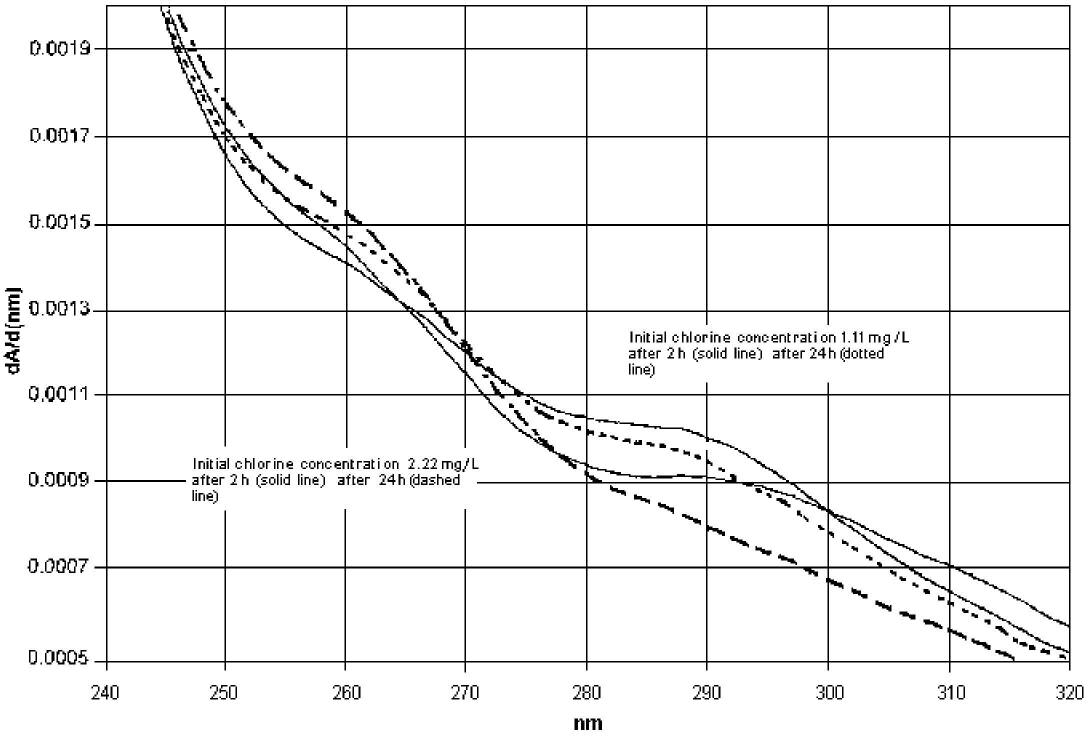

**FIGURE 2.** The change in first derivative UV spectra of chlorinated lakewater, at initial chlorine concentrations of 1.11 and 2.22 mg/l, measured after 2 and 24 h, respectively.

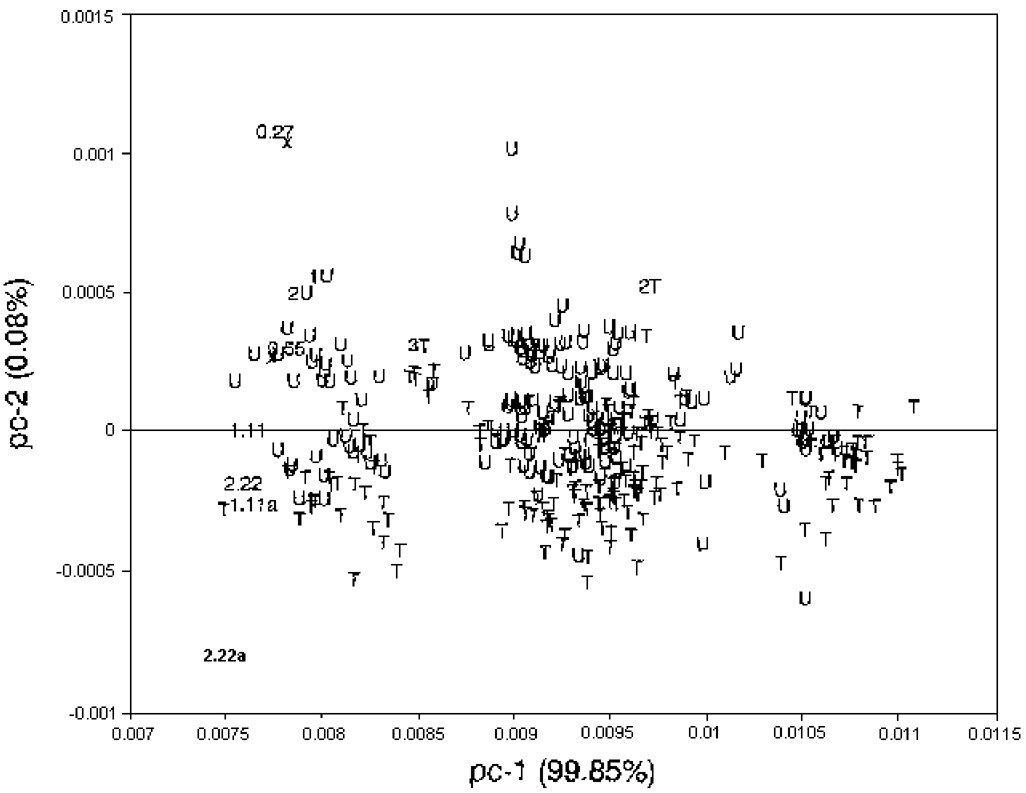

FIGURE 3. Projection of the first derivative UV spectra on the plane of the first two principal components. Positions of samples are indicated by category: large-volume user (U), town (T). Samples from model experiments are marked by the initial concentration of chlorine: (0.27, 0.55, 1.11, and 2.22), when measured after 2 h, and by 'x', 'x', 1.11a, and 2.22b, when measured after 24 h. The amounts of original sum of squares, captured by the principal components are indicated on the axes. First derivative spectra of samples (1U, 2U, 1T, 2T, 3T) are shown in Fig. 5.

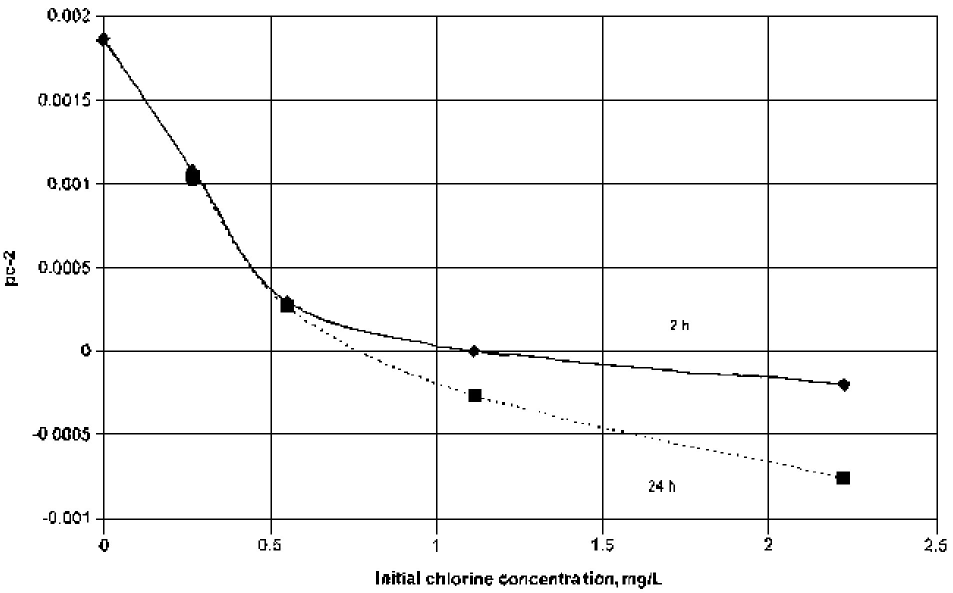

FIGURE 4. Scores on the pc-2 axis of first derivative spectra of lakewater, chlorinated in the laboratory, plotted against the initial chlorine concentration. The spectra were scanned 2 and 24 h after chlorination.

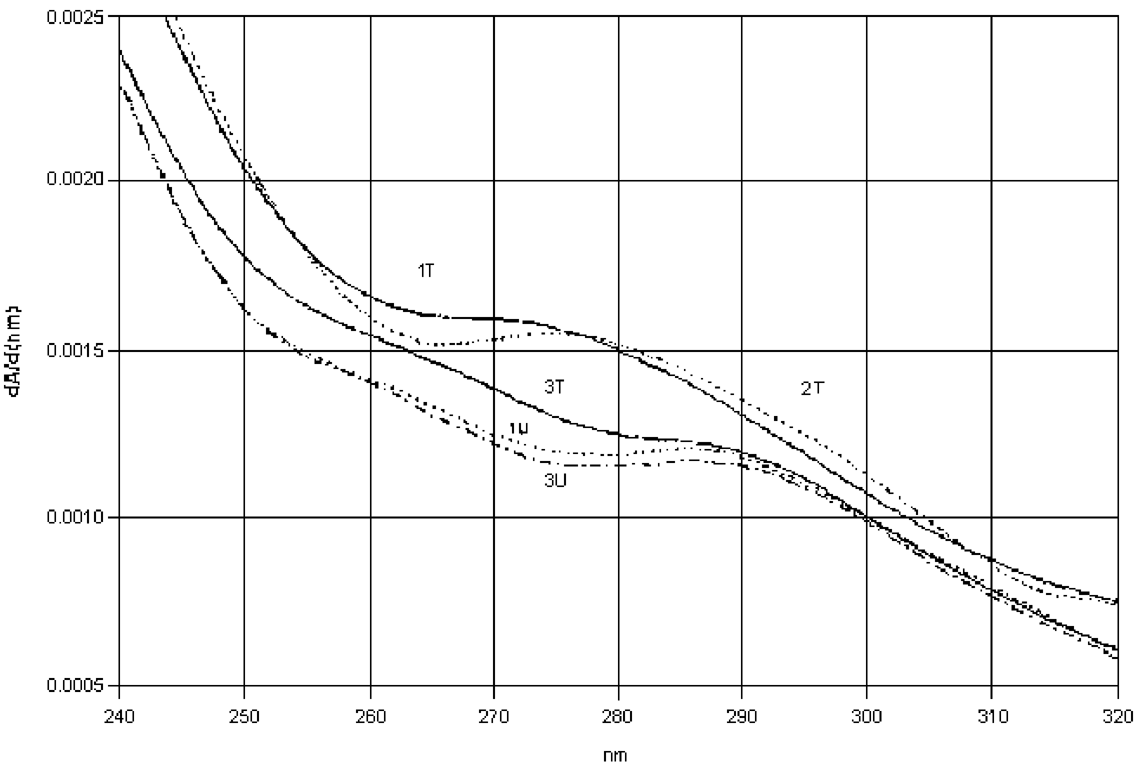

**FIGURE 5.** First derivative UV spectra of samples 1U, 2U, 1T, 2T, and 3T (see Fig. 3), which received little chlorination.

occasions, little or no chlorination was performed at the lake and that this water also entered the town system. Such occurrences are potentially dangerous to public health.

## ACKNOWLEDGMENTS

I thank Ms. Marilynn Rudi for editorial comments.
